# The development and implementation of trauma, posttraumatic growth and trauma-informed rehabilitation course for psychiatric personnel-case study

**DOI:** 10.1192/j.eurpsy.2023.2194

**Published:** 2023-07-19

**Authors:** Y. Mazor, A. Ezra, N. Hadas-Lidor

**Affiliations:** 1 Ono Academic college, Kiryat Ono; 2 School of social work and welfare, Hebrew university of Jerusalem, Jerusalem; 3 School of social work, Ashkelon Academic college, Ashkelon, Israel

## Abstract

**Introduction:**

The National School for Training in Mental Health Rehabilitation and Recovery at the Ono academic college operates under the auspices of, and is funded by, the Division for Mental Health Rehabilitation of the Israeli Ministry of Health. Intended to hone the quality of services provided, it offers numerous training courses, that target various populations associated with and working in the field of mental health recovery, among them clients and their family members, as well rehabilitation professionals and support personnel. The novel course of trauma and posttraumatic growth (PTG) was developed and aimed at supporting recovery, coping strategies and ultimately PTG through the lenses of both recovery and trauma informed care for psychiatric and support personnel, professionals with lived experience, and family members. It is built of eleven six hours long sessions with lectures, in-vivo assignments, and group supervision.

**Objectives:**

Portray the development, implementation, and outcomes of the course from psycho-educational, clinical, social, and personal perspectives of students as well as the course’s developers.

**Methods:**

A case study of the course that will be portrayed through qualitative anonymous testimonials from students’ surveys and assignments, as well as the analyses of processes that the course underwent through the years.

**Results:**

The course has several dimensions that allow the integration between trauma, recovery, and PTG in psychiatric rehabilitation: the integration between academia and research-based knowledge with experiential knowledge; integration between the perspectives of PTG and recovery; and, integration between social and cultural perspectives and person-centred care. In addition, the course promotes trauma-informed models in psychiatric rehabilitation; offers new perspective and implantation to psychiatric recovery-oriented tools so as to promote PTG; and enhances social support and cohesion within mental health services and personnel.

**Conclusions:**

The current case study portrays the unique processes of knowledge development, implementation, and training in psychiatric rehabilitation personnel, supported staff and people with lived experience. The course brings a focus on PTG as a promising addition to the trauma-informed approach in psychiatric rehabilitation, which is only scarcely linked to recovery.

**Disclosure of Interest:**

None Declared

